# ISAC based seamless handover solution for SAGIN in high mobility environments

**DOI:** 10.1038/s41598-025-29697-6

**Published:** 2025-11-30

**Authors:** Madeeha Aman, Muhammad Zubair, Riad Alharbey, Ghani Ur Rehman, Sajid Ullah Khan, Ali Daud

**Affiliations:** 1 Department of Computer Science, University of the Punjab, Lahore, Pakistan; 2 Department of Computer Science and Bioinformatics, Khushal Khan Khattak University, Karak, Pakistan; 3 Department of Information Systems and Technology, College of Computer Science and Engineering, University of Jeddah, Jeddah, Saudi Arabia; 4 Department of Information Systems, College of Computer Engineering and Sciences, Prince Sattam bin Abdulaziz University, Alkharj, Kingdom of Saudi Arabia; 5 Faculty of Resilience, Rabdan Academy, Abu Dhabi, United Arab Emirates; 6 Heriot Watt University (Zhubanov Campus), Aktobe, , Kazakhstan

**Keywords:** SAGIN, High-mobility, Handover, ISAC, Space-air-ground, 5G, 6G, Energy science and technology, Mathematics and computing

## Abstract

Seamless and uninterrupted connectivity within high-mobility environments (e.g. aerospace and high-speed terrestrial transportation) drives a critical demand for Space-Air-Ground Integrated Networks (SAGIN). In such dynamic environments, conventional handovers lead to frequent loss of connection, higher latency, and diminished quality-of-service (QoS). A new high-mobility handover procedure for SAGIN based on the concept of Integrated Sensing and Communication (ISAC) is introduced. We suggest a new method using real-time environmental perception to improve the decision-making of hand over, reducing the frequency of handover, and balancing the spatial, air and ground network resource allocation. It combines dynamic priority scheduling and a cross-layer communication protocol into a general handover procedure for seamless mobility between network layers. MATLAB-based simulations show that, when compared to existing ground-based and satellite-assisted handover methods, the overhead of LEO-enabled handovers often results in a substantial reduction in latency while simultaneously providing enhanced network throughput with minimum packet loss. The results show that our approach is appropriate to preserve an agreement on the quality received in highly dynamic environments, opening perspectives with novel scientific questions for SAGIN and high-mobility networks.

## Introduction

The requirements for maintaining seamless connectivity in high-mobility scenarios are expected to intensify as we transition towards sixth-generation (6G) wireless communication networks. SAGINs are appearing as a promising solution for ubiquitous high-speed communication services delivery to stationary and mobile terminals (aircraft, UAV, satellites, terrestrial vehicles)^1,2^. Yet, these networks must overcome the key challenge of holding continuous connection reliability throughout services, especially during the handover period under high-transit conditions, like while a drone or satellites discover their path^3,4^. The emergence of ISAC technology is a very promising approach to meet these challenges by integrating the communication and sensing functionality so that both network performance and sensing accuracy in such dynamic environments can be enhanced^1,5,6^.

In traditional systems, the handover mechanisms tend to induce high latency as well as packet loss due to which they are not desirable for high-speed mobile scenarios. Since the nodes in SAGINs such as UAV and satellites move quickly, they require frequent handover between different network segments. Present handover designs are primarily communication-centric, meaning they cannot effectively optimize for the fast morphing nature of high-mobility scenarios. On the other hand, ISAC-based handover mechanisms present a chance to merge these two functionalities as they facilitate seamless adjustment of the network regarding mobility situations via predictive models and instantaneous state information from constantly streamed environment^7^. Through ISAC, the network can predict a handover event using real-time information and reduce latency and service interruption^8,9^.

ISAC can not only maximize the efficiency of spectrum usage by utilizing the same frequency band for both sensing and communication at the same time but also enhance those detection capabilities that are essential in high-mobility scenarios. For instance, ISAC systems are capable of performing detection, tracking and prediction for the trajectory of mobile nodes to facilitate more efficient handover procedures by adjusting network parameters in advance. Real-time evaluation of the environmental information such as location, velocity and flight path of cars and UAVs will make advance handover possible, therefore enhancing performance of integrated networks and avoiding the handover failure^1,5,10^.

### Other handover strategies in SAGINs

One of the key problems in SAGINs is that of the handover when a user’s connection is passed from one node of the network (e.g., satellite, aircraft, or ground station) to the other. Numerous research studies suggest new handover schemes to improve the overall network performance.

The dynamic handover software defined transmission control scheme^11^ approach addresses the management of handover in SAGINs using software defined networking (SDN). With this new addition, the existing approach can dynamically handle the handover management based on the changing network and user conditions ensuring efficient data transmission over the networks.

The evolutionary game based vertical handover strategy^12^ offers a more sophisticated solution via a game-theory model used to optimize the handover decision process. It takes into account several elements, including but not limited to, network congestion and end-user preferences, which enables a better decision-making process while handing over connections in heterogeneous networks (satellites, aircraft, ground stations, etc.)

The authors in^13^ propose a solution for Low Earth Orbit (LEO) satellite networks using load balancing and carrier-to-noise ratio (CNR) based handover algorithm. This algorithm measures the CNR between satellites and users to outline the optimal handover point, thus mitigating latency and signal reduction, especially present in dynamic conditions where LEO satellites are constantly moving.

Low-delay secure handover is one of the significant factors of SAGINs, where security and low delay are the two critical factors for handover management. The low-delay secure handover^14^ is a fast secure handover approach to realize a low delay of data transmission during handovers. This research emphasizes techniques to secure the integrity and confidentiality of user data in the handover process, which is critical for military or sensitive communications.

### Resource management and mobility management in SAGINs

In a multi-tier environment like SAGINs, where communication is via three different layers namely space, air and ground, efficient resource management is vital for optimizing the performance of the network.

The authors investigate the integrated computation resources in SAGINs^15^, which discusses the resource management. Joint communication and computing is a paradigm for distributing computational tasks to the edge devices or satellites, which can yield performance improvement in the ground network as well as in the overall system. Such resource management strategy improves efficiency, lower latency, and provide better quality of service (QoS) for users. Another essential building block for SAGINs is integrated sensing and communication. The authors in^16^ proposes a strategy that combines sensing technologies, such radar and satellite-based positioning systems, with communication networks for mobility management. This approach will help manage user mobility in contexts in which location and trajectory data are important for handover and resource allocation purposes.

### Emerging trends in SAGINs

As the field of SAGINs continues to develop, several emerging trends are shaping the future of handover management and resource optimization.

As the 6G era approaches, the integration of space, air, and ground networks will become even more critical. This research^17^ relates to 6G technologies enabling holographic communication, and ultra-high-speed lower-latency applications with SAGINs with ultra-high speed capability, and will support autonomous systems. The authors also emphasize the use of AI to enhance network performance through predictive handover management, dynamic spectrum allocation, and automated resource optimization. The authors in^18^ focuses on target handover in the context of distributed integrated sensing and communication in SAGINs. Using machine learning and artificial intelligence will allow the system to learn in real time from user behavior, network states, and environmental factors and predict and optimize handovers. This system would require less human intervention, and users would have a much more seamless experience.

In this respect, by using ISAC-based predictive sensing and communication techniques, a new ISAC handover procedure is proposed to improve handover performance in SAGIN. It provides a very simple, flexible, and fault-tolerant network that is capable of bearing high-speed mobility^19^. This strategy is proposed to further reduce handover latency and ensure link connectivity for high speeds if we keep into account the multidimensional aspects involving not only communication but also sensing metrics^8,20–22^. The followings are the main contributions of this article.

- Improved the handover performance in Space-Air-Ground integrated Networks (SAGIN).
- Designing of new procedure, process for handover, and litigation in handover latency and better link stability.
- Improved the overall network performance statistics and provide advanced decision making and scheduling methods.

The rest of this paper is organized as follows: A review of related work on ISAC and handover procedures in wireless networks is presented in “Literature review”. In “Proposed system” the ISAC-based handover mechanism is proposed. Numerical Analysis & Evaluation is discussed in “Numerical analysis and evaluation”, the simulation results are presented in “Simulation and results”, In “Conclusion” the article is concluded and finally a summary of future research directions and challenges is provided in “Current challenges and future work”.

## Literature review

### Seamless handover in satellite and aerial networks

The dynamic nature of high-mobility systems (satellite and aerial networks, in particular) poses a significant challenge to handover. Especially for LEO satellite networks, handovers occur frequently due to the fast movement of satellites across the sky. Existing handover schemes meant for conventional terrestrial networks are not able to effectively meet these specific requirements of LEO networks. To address these challenges, the authors in^23^ describe a user-oriented handoff scheme for ultra-dense LEO satellite networks where the storage capabilities of satellites are leveraged to buffer user data over multiple satellites at the same time. We use this method to achieve smooth handovers by making the terrestrial user always connected with the best link quality satellite, so we can minimize handover delay and maximize the overall communication quality. Also, the work presented in^24^ proposed a QoE-driven intelligent handover mechanism for mobile satellite networks (MSNs). By using pre-calculated service time and communication channel resources, this method significantly improves the user experience by lowering handover failure rates while improving end-to-tend latency performance. This approach combines the analysis of user mobility and satellite trajectories to transfer the user for the best-suited satellite, such that transmission loss is.

### The role of ISAC in high-mobility handover procedures

Integrated Sensing and communication (ISAC) is a disruptive technique for next-generation wireless networks that opens up possibilities to leverage dual-functionality (i.e., sensing and communication operations simultaneously). ISAC allows fast in-situ sensing that can be applied to improve mobility management and handover decisions for both high-mobility cases expected e.g. In^25^, the authors investigate the concepts of intelligent reflecting surface (IRS)-assisted ISAC in high-mobility systems with OTFS modulation to combat the severe Doppler effects. By integration of sensing data, link quality can be more efficiently managed and subsequently an optimized handover decision can be carried out. This way enables a resilient communication in high-mobility environment.

In ISAC-based handover mechanism, the authors have proposed a machine learning based methodology that works towards bringing more intelligent handover mechanisms in place so that communication systems could adapt based on changing environment. The user association and handover problem with mmWave is also addressed in^26^, which we consider an important aspect of learning-based algorithms when they deal with resource management, showing how RL techniques are well-suited to adapt the handover strategy based on real-time network status. It can help network controller to anticipate the best moment and target for handover by incorporating RL in ISAC-based systems which lowers failure rate of Handover that leads to better overall performance of Network.

### Joint communication, sensing, and computing in SAGIN

Achieving the complexity of SAGIN environments critically requires the convergence of communication, sensing, and computing functionalities. In^27,28^ an integrated system architecture is introduced which integrates these three key components to enable seamless handover between network layers. To achieve this, their architecture of mobile edge computing (MEC) at the edges locally processes massive volumes of sensing data instantaneously to discover user positions and link quality dynamically for precise handover decisions follow these conditions, such as immediate users positions, the predicted ones, and network load changes. By combining these elements, a better allocation of resources can be reached and the maintenance of connection continuity will have better outcomes especially in scenarios with high mobility and handovers.

In addition, ISAC benefits significantly from the utilization of STAR-RIS that can simultaneously perform as a transmitter and reflector. ATN-MAR: STAR-RIS-based ISAC Systems for Enhanced Communication and Sensing Performance Enhanced Communication and Sensing Performance with STAR-RIS-based ISAC Systems Response of Target^29^ propose a STRAW-RIS-based ISAC system that improves both communication and sensing performance by adaptively varying the direction of beamforming associated with the handover opportunities in order to improve link quality. They have exploited the special features of STAR-RIS (bilateral reflection and transmission) to design a system that can provide continuous handover management without requiring line-of-sight communication, which is impossible for conventional methods.

### Machine learning and intelligent handover mechanisms

In a bid to optimize decision making in dynamic network scenarios, machine learning and artificial intelligence are gaining momentum as integrated components of handover mechanisms. In particular, reinforcement learning has been effective in cases where real-time adaptation is important. Mobile satellite networks are another use case that has seen promising work leveraging reinforcement learning for predicting handover factors and optimizing QoE^24^. The system predicts the availability of satellite links and determines the optimal handover target through the formulation of a multi-dimensional quality-of-service (QoS) function which is optimized using reinforcement learning methods, combining bandwidth, service time and user mobility patterns.

In a similar vein,^30^ investigates mobility-aware seamless handover in software-defined heterogeneous networks (SDHetNets) where the authors again leverage multi-path transmission control protocol (MPTCP) to ensure service continuity when users move from one base station to another. By accurately predicting the mobility pattern of users and allocating network resources ahead of time, their proposed system can diminish handover delays and avoid the ping-pong effect scenario where users switch back-and-forth between networks without successfully connecting. To this end, it emphasizes mobility prediction as well as intelligent resource management to guarantee seamless connectivity in high mobility scenarios.

ISAC can be a key enabler to support SAGIN. ISAC can by facilitating integration with handover procedures, which is critical for maintaining connectivity and seamless service continuity in high-mobility environments such as SAGIN. With the aid of real-time sensing data along with machine learning algorithms and besides intelligent reflecting surfaces(IRS), ISAC based systems enables more intelligent and smart linked handover decisions to minimize service interruptions as well as pivotal in enhancing general network performance. The reviewed literature emphasizes the need for an adaptive, intelligent handover mechanism invulnerable against unique circumstances challenges in high-mobility environments especially for satellite and aerial networks.

## Proposed system

In this paper, we design a handover procedure with ISAC for SAGIN which is capable of improving efficiency as well as the performance of the process of handover in high-mobility environments.

### System architecture and modules

**Fig. 1:**
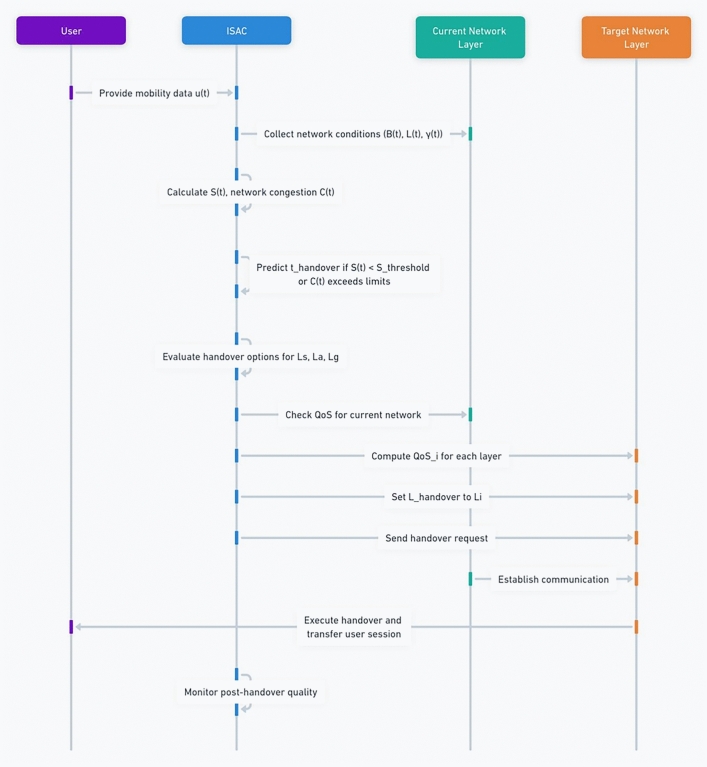
Proposed handover procedure.

The proposed handover system is composed of interconnected modules, each playing a key role in the decision-making process. The modules are designed to operate collaboratively, exchanging real-time data to ensure seamless handover management in high-mobility scenarios. The modules and their interconnections are as follows:

- ISAC-enhanced sensing module: this module plays the primary role of gathering environmental and network data in real-time. It continuously captures key attributes such as the location (*u*(*t*)), velocity (*v*(*t*)) of users, signal strength (*S*(*t*)), and network congestion levels (*C*(*t*)) from devices like GPS receivers, accelerometers, and signal strength sensors.
  Interconnection: the ISAC module feeds real-time sensing data into the Multi-Layer Handover Decision Engine. The output from the ISAC module (predicted handover points based on the user’s mobility and network conditions) provides critical information to optimize the network layer selection for handover.
- Multi-layer handover decision engine: this engine is responsible for analyzing the data collected by the ISAC module and determining the best network layer for handover (space, air, or ground). It evaluates Quality of Service (*QoS*) metrics, such as Signal-to-Noise Ratio (*SNR*), Available Bandwidth (*B*), and Latency (*L*) for each network layer. It then selects the network layer that will provide the best performance for the user based on the calculated QoS for each layer.
  Interconnection: the decision engine receives mobility-related data from the ISAC-Enhanced Sensing Module, user priority data from the Dynamic Priority Scheduling Module, and network layer status from the Cross-Layer Communication Protocol. These inputs help it to assess and determine the most suitable network layer for handover.
- Dynamic priority scheduling: this module assigns priority levels to users based on their mobility profiles (speed and location). Users moving at higher speeds or requiring more stable connections (e.g., aircraft or UAVs) are prioritized to reduce handover latency. The priority score for each user is calculated based on their velocity and signal strength.
  Interconnection: the priority scores are fed into the Multi-Layer Handover Decision Engine, which uses this information to prioritize handovers for faster users, ensuring that high-priority users are transitioned to a new network layer before signal degradation affects their connection.
- Cross-layer communication protocol: this protocol ensures seamless communication between the space, air, and ground layers. It coordinates the handover request and facilitates the exchange of control signals across network layers. This module also enables real-time coordination between the ISAC-Enhanced Sensing Module and the Multi-Layer Handover Decision Engine to quickly dispatch commands for handover execution.
  Interconnection: the cross-layer communication protocol interacts with both the ISAC Module for data exchange on the user’s movement and network conditions, and the Multi-Layer Handover Decision Engine to confirm the selected network layer and trigger the actual handover. It also ensures that the handover command is received by the target layer and that the session transfer occurs without disruption. The handover procedure is depicted in Fig. 1.

### Proposed handover procedure

The handover process follows a structured workflow:

1. Environmental sensing with ISAC module:
  The Integrated Sensing and Communication (ISAC) module constantly senses the user’s mobility status, network condition, and environmental factors.
  Key metrics like user location, speed, signal strength of the user and others for network congestion are collected in real-time.
2. Prediction of potential handover points:
  Based on the collected data, the ISAC module predicts when and where it may be necessary to transfer network connections. This requires pinpointing the places where the strength of the signals are weakening or where there is a clogging in networks.
  The module predicts the perfect moment to start the handover process to reduce unnecessary handovers.
3. Multi-criteria decision analysis for optimal layer selection:
  Based on the absorbed information from each of these layers, the MSS-MHDE (Multi-Layer Handover Decision Engine) analyzes potential handover candidates within space, air and ground spectrum by using its own methodology.
  It performs a comparison among different layers by comparing parameters such as SNR, unutilized bandwidth, and end-to-end delay to choose an optimal layer of network for the handover.
  The decision engine picks which layer best to keep the quality of service and connection to users.
4. Priority based scheduling of users:
  Users are prioritized based on their mobility profiles (speed, and signal conditions).
  To facilitate seamless connectivity, high-mobility users (such as users in air crafts or fast vehicles) are prioritized for handovers.
  Dynamic Priority Scheduling: User priority levels are assigned in a dynamic manner to serve multiple users that require handovers at the same time.
5. Cross-layer communication to trigger execution:
  The corresponding handover decision is then sent to the respective network layers (space, air and ground) using the Cross-Layer Communication Protocol.
  This protocol coordinates the handover process between the source network layer and the chosen target network layer.
  The handover is triggered and the target network layer gets ready to take over the connection.
6. Seamless transfer and post handover monitoring:
  It is conducting a handover, which means, we switch user connection from one layer of a network to this target layer with as little disruption as possible.
  The network guarantees that only active sessions, data streams and user preferences are seamlessly transitioned.
  Once this information is passed on to the ISAC module, the user layer goes on with monitoring the quality of that connection without needing multiple handovers or switching reconnects and works accordingly based on new network calls separating itself from the previous ones in real-time called another network layer.
  In case more handovers are needed, the process repeats subject to real time environmental conditions.

**Fig. 2:**
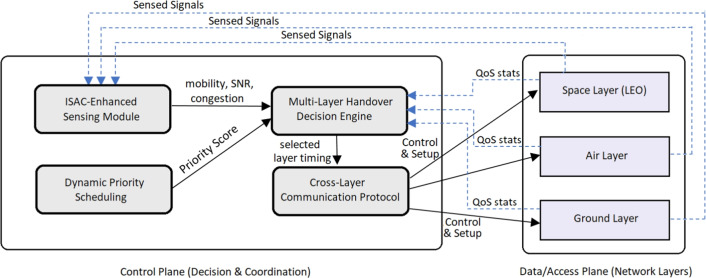
Cross-layer interactions in the proposed ISAC-based handover system: ISAC and priority modules feed the Multi-layer handover decision engine; the cross-layer protocol executes the handover across space, air and ground layers, while QoS/ Sensing feedback continuously updates the controller.

The Cross-Layer interactions in the proposed ISAC-based handover system is shown in Fig. 2.

### Mathematical model and notations

- Notations table:
  Table 1 presents the notations used in the proposed system.
- Optimal handover time: the handover latency is determined by minimizing a linear combination of the decrease in signal strength and congestion of the network. We can use gradient descend methods to solve for the minimization problem, and we can compute the gradient given in Eq. (1):
  1 $$\begin{aligned} \frac{d}{dt}\big [ S(t)- \Delta S. d(u(t)), AP)+\lambda . C(t)\big ]=0 \end{aligned}$$
  which gives us the optimal period where the signal degradation rates exceed the networks congestion values.
- Layer selection based on QoS: QoS optimization can be modeled as MCDA (Multi Criteria Decision Analysis) problem. Normalizing the SNR, bandwidth and latency to their thresholds, the decision engine chooses an optimal layer that is handed over. Once these parameters have been extracted, they contribute to an overall utility function, and the handoff occurs over the layer where the global utility is maxed.

**Table 1:** Notations table.

Notation	Description
*u*(*t*)	User mobility profile at time *t*
*v*(*t*)	User velocity at time *t*
*S*(*t*)	Signal strength at time *t*
*C*(*t*)	Network congestion at time *t*
$L_{handover}$	Selected network layer for handover (space, air, or ground)
$QoS_{i}(t)$	Quality of Service for network layer *i*
*P*(*u*(*t*))	Priority score for user based on mobility profile

**Fig. 3:**
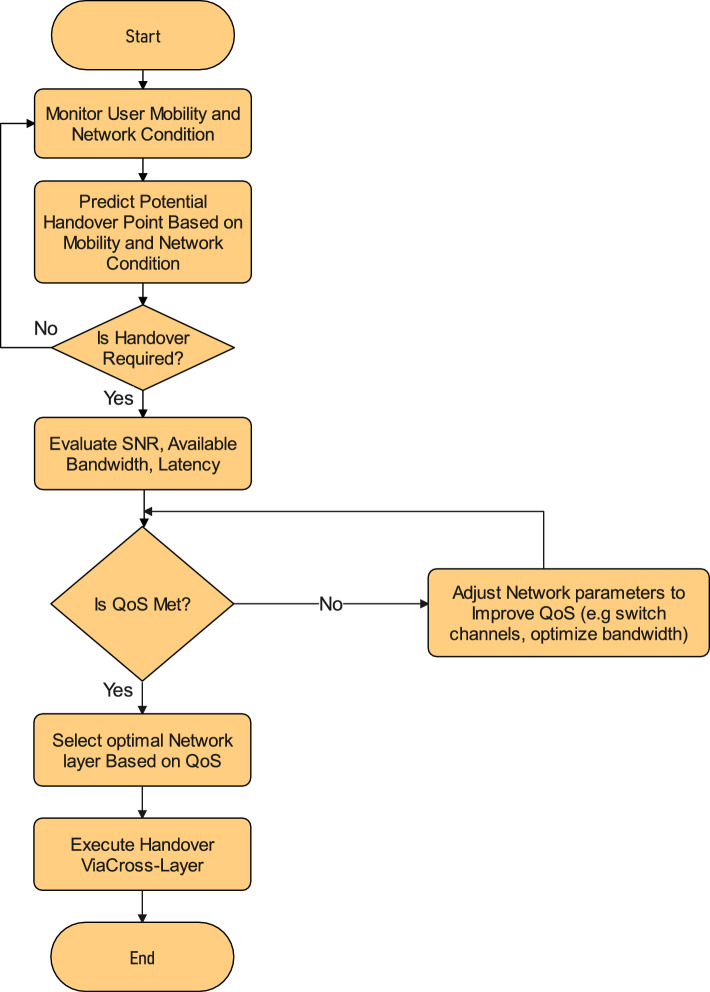
Flowchart of the proposed scheme.

Based on the characteristics of ISAC in SAGIN, we propose a novel handover process and procedure. It focuses on high-mobility users and tries to enhance network efficiency with small interruption by optimizing the handover decision. To do so, the paper employs ISAC-related sensing, multi-layer handover decision-making engine, dynamic priority scheduling and a cross layer communication mechanism. The flowchart of the proposed scheme can be seen in Fig. 3.

#### ISAC-enhanced sensing module

In the ISAC-enhanced sensing module, real-time data from various sensing devices is continuously monitored. These devices include GPS receivers for location tracking, accelerometers for velocity and direction measurements, and signal strength sensors to detect the quality of communication channels. Additionally, congestion sensors and network traffic analyzers are employed to evaluate the congestion level at the current access point (*AP*). Key parameters for predicting handover points include the location (*u*(*t*)), velocity (*v*(*t*)) of the user, signal strength (*S*(*t*)), signal degradation rate $(\Delta S)$, and the congestion level (*C*(*t*)) of the serving *AP*. The ISAC module utilizes these parameters to predict the optimal handover time by analyzing user movement trends, network conditions, and signal fluctuations. This prediction allows for the proactive adjustment of the network layer, reducing unnecessary handovers and enhancing overall network performance. Let:

- *u*(*t*) be the location of the user at time *t*,
- *v*(*t*) be the velocity of the user,
- *S*(*t*) represent the signal strength from the current access point (AP),
- $\Delta S$ be the signal degradation rate, and
- *C*(*t*) be the congestion level of the serving *AP*.

The ISAC module calculates the optimal handover time $t_{handover}$ by solving the following optimization problem as given in Eq. (2):

2 $$\begin{aligned} t_{handover}=arg. min_{t}\big \{S(t)-\Delta S. d(u(t), AP) + \lambda .C(t)\big \} \end{aligned}$$

where *d*(*u*(*t*), *AP*) is the distance between the user and the current *AP*, and $\lambda$ is a weighting factor that adjusts the influence of network congestion on the handover decision.

#### Multi-layer handover decision engine

The handoff decision engine works on all three SAGIN air, space, and ground layers, based on the user mobility profile/condition of the network layer, it selects a best suited network layer. Consider three network layers: space $L_{s}$, air $L_{a}$, and ground $L_{g}$. In the Multi-Layer Handover Decision Engine, the handover decision is made by evaluating a set of Quality of Service (QoS) metrics for each network layer (space, air, and ground). These metrics are critical in ensuring the selection of the most appropriate layer to hand over the user’s connection. To make an informed decision, the engine evaluates the following QoS metrics:

- Signal-to-noise ratio (SNR), $\gamma _{i}(t)$: a higher SNR indicates better link quality and is preferable for handover. A low SNR may indicate the need for handover to a more stable layer.
- Available bandwidth $B_{i}(t)$: adequate bandwidth is essential for ensuring that the user maintains a high data rate during the handover process. If the available bandwidth is insufficient, the decision engine will prioritize moving the user to a layer with more available bandwidth.
- Latency $L_{i}(t)$: low latency is critical for real-time services. If the latency exceeds the acceptable threshold, the user should be handed over to a layer that can provide a more responsive connection.

1. Thresholds for QoS metrics: Each QoS metric is evaluated against a predefined threshold to determine whether the current network layer is suitable for maintaining the connection. These thresholds are used as criteria to decide when a handover is required. Specifically, the thresholds are as follows:
  SNR threshold ($\gamma _{(threshold)}$: The minimum acceptable value for SNR below which the connection quality is considered poor. A handover is triggered if the SNR drops below this threshold.
  Bandwidth threshold $(B_{max})$: the maximum acceptable value for bandwidth. If the available bandwidth is below $B_{max}$, indicating that the current layer cannot support the user’s data rate, the user should be handed over to a more capable layer.
  Latency threshold $(L_{max})$: the maximum allowable latency that can be tolerated for seamless connectivity. If *L* exceeds $L_{max}$, a handover is initiated to reduce delay.
2. Multi-criteria decision analysis (MCDA) for handover: to make the handover decision, the system employs Multi-Criteria Decision Analysis (MCDA), where the goal is to select the optimal network layer based on the QoS metrics. The MCDA framework evaluates the performance of each layer by considering the following utility function for each layer $L_{i}$ at time *t* is given in Equation (3):
  3 $$\begin{aligned} QoS_{i}(t)=w_{1}. \frac{\gamma _{i}(t)}{\gamma _{threshold}}+w_{2}.\frac{B_{i}(t)}{B_{max}}-w_{3}.\frac{L_{i}(t)}{L_{max}} \end{aligned}$$
  where $w_{1}, w_{2}, w_{3}$ are weighting factors that are crucial in determining the relative importance of each metric in the decision-making process. For example, in a video streaming scenario, latency might be more critical, so *w*3 would be given a higher value. In a data transfer scenario, bandwidth may be more important, leading to a higher *w*2. $\gamma _{threshold}$ is the SNR at time *t* for layer *L* *i*, $B_{i}(t)$is the available bandwidth for layer *L* *i*, $L_{i}(t)$ is the latency for layer *L* *i*, and $\gamma _{threshold}$, $B_{max}$ are the threshold values for SNR, bandwidth, and latency. The utility function evaluates each network layer (space, air, ground) based on how well it satisfies the QoS requirements. The layer with the highest utility score is selected for the handover decision.
3. Handover decision: Once the utility scores for each layer are calculated, the layer with the highest utility score is selected for handover and is given by Equation (4):
  4 $$\begin{aligned} L_{handover}=arg. max_{i}(QoS_{i}(t)) \end{aligned}$$
  where $L_{handover}$ is the selected network layer (space, air, or ground) for the user to be handed over to. For example, if the SNR in the air layer is high, but the bandwidth in the ground layer is more suitable for high-throughput applications, the MCDA approach will evaluate the QoS metrics based on the weight factors assigned to each. If the ground layer has a better overall QoS score, despite the air layer having a high SNR, the user will be handed over to the ground network.

#### Dynamic priority scheduling

Certain users need to be served at a higher priority during the handover process in high-mobility environments^26^. For instance, ultra-high-speed flying users will perform signal arrival losing quickly, thus leading to fast handover. In dynamic and high-mobility environments, it is crucial to assign priorities to users to ensure that high-priority users are served with the least disruption during handover. The dynamic priority assignment considers the user’s mobility profile (e.g., velocity, signal strength) and assigns a priority score to each user. This prioritization is vital for determining which users should be handed over first, especially when multiple users are competing for resources.

1. Dynamic priority assignment: Users are assigned dynamic priority scores based on two primary factors:
  Velocity (*v*(*t*)): The speed at which a user is moving, as faster-moving users require quicker handovers to avoid service disruption.
  Signal strength (*S*(*t*)): The current signal strength from the access point (AP), where weaker signals indicate a higher likelihood of connection failure and thus should be prioritized for handover.
  The priority score *P*(*u*(*t*)) for a user is given in Equation (5):
  5 $$\begin{aligned} P(u({t}))=\alpha . v(t) + \beta . \frac{S(t)}{S_{min}} \end{aligned}$$
  where *v*(*t*) is the velocity of the user at time *t*, *S*(*t*) is the signal strength at time *t*, $S_{min}$ is the minimum acceptable signal strength to maintain the current connection, and $\alpha$ and $\beta$ are weight factors that control the influence of velocity and signal strength on the priority score. Higher priority is given to users with greater velocity (i.e., users moving faster, such as aircraft) or those with weak signal strength, ensuring that these users are handed over first to prevent service disruption.
2. Scheduling algorithm: The dynamic scheduling algorithm governs the handover process by ensuring that users with higher priority are given preference. The scheduling process works as follows:
  Input: The algorithm takes the list of users in the handover zone, their current priority scores, and network layer conditions (signal strength, available bandwidth, and network congestion).
  Priority calculation: For each user, the priority score *P*(*u*(*t*)) is calculated based on the formula above. The user with the highest priority score is selected for handover first. This ensures that users requiring immediate attention (such as fast-moving vehicles or UAVs) are processed before others.
  Resource allocation: Once the user with the highest priority is selected, the algorithm checks the available resources in the next network layer (space, air, or ground). If the resources are sufficient, the handover process is triggered for that user. Otherwise, the next highest-priority user is considered.
  Handover execution: After the highest-priority user is handed over, the system proceeds to the next user in the priority list, ensuring that users with higher mobility or weaker signals are served first. The system repeats the process until all users in need of handover are successfully transferred.
  The dynamic priority scheduling algorithm aims to minimize handover delay and packet loss by prioritizing users based on real-time conditions, ensuring smooth transitions even in high-mobility scenarios.
3. Example use case: Consider a scenario where a fast-moving aircraft and a slow-moving vehicle are in the same network area. The aircraft will have a higher priority due to its higher velocity, meaning it will be handed over to the next available network layer before the vehicle. This prioritization minimizes handover failures and ensures that users in critical mobility conditions (e.g., high-speed aircraft) retain seamless connectivity.

#### Cross-layer communication protocol

Among the contributions of our proposed approach is the idea of cross-layer communication protocol across the space, air and ground layers so that we can get fast and seamless handovers. It is used to coordinate signaling across network layers to control hand over process. The following are some steps in the protocol:

- Sensing data collection & handover time prediction via ISAC module.
- QoS is evaluated for all layers by the Decision Engine, which selects the final layer for handover.
- Then, utilizing the cross-layer protocol, a handover request is sent to the appropriate layer.
- The new AP serves a new session and migrates the user session to this new layer of network.

By keeping all the layers of the network aware of the mobility state, a handover request can be answered very quickly-if so desired by the network, this protocol reduces the time it takes for a handover to occur. The Cross-Layer Communication Protocol is a critical component of the proposed handover procedure, enabling seamless communication across the three key layers of the network: space, air, and ground. This protocol ensures that handover operations are managed efficiently and that all network layers are synchronized to avoid service disruption. Below is a detailed breakdown of how this protocol operates:

1. Role and purpose: The primary role of the Cross-Layer Communication Protocol is to coordinate the signaling between the space, air, and ground layers during the handover process. This coordination is essential to ensure that the handover decision made by the Multi-Layer Handover Decision Engine is acted upon quickly, and that the transition between layers occurs without significant delays or interruptions in service.
2. Key functions:
  Signaling coordination: The protocol ensures that when a handover decision is made, handover signals are sent across all relevant layers of the network (space, air, and ground). This signaling process is synchronized, so the handover command reaches the appropriate target layer, and the source layer is informed that the user session is being transferred.
  Real-time data exchange: During the handover process, the protocol facilitates the real-time exchange of key data between layers, such as:
    User mobility information: Current location, speed, and direction.
    Network conditions: Signal strength, congestion levels, and available bandwidth for the space, air, and ground layers.
    Handover status: Updates on the current handover progress and any failures.
    This ensures that each layer has up-to-date information about the status of the user and the network conditions, allowing for quick adjustments as needed.
3. Handover execution: Once a handover decision is made, the handover request is sent to the target layer using the cross-layer communication protocol. This request contains essential information such as:
  (a): The user’s current session information (to ensure continuity in service).
  (b): The selected target layer (whether it is the space, air, or ground network).
  (c): The handover timing (to ensure the transition occurs at the optimal moment). Upon receiving the request, the target layer prepares to take over the connection, while the source layer starts the handover process, ensuring minimal disruption to the user’s connection.
4. Seamless layer coordination: The protocol ensures that the layers work together in a seamless manner, reducing the time needed for handover and preventing issues like ping-pong effects (when users switch back and forth between layers without successfully connecting). The protocol achieves this by:
  Synchronizing layer activities: Ensuring that each layer is ready to assume control before the handover actually takes place.
  Controlling layer access: Managing which layer is responsible for the user’s session at any given time, preventing conflicts and delays.
5. Steps involved in cross-layer communication: The steps involved in cross-layer communication during the handover process are as follows:
  Handover prediction and decision:
    (a): The ISAC Module detects potential handover points based on the user’s mobility and network conditions and informs the Multi-Layer Handover Decision Engine.
    (b): The engine calculates the optimal handover layer (space, air, or ground) based on QoS metrics and sends this decision to the Cross-Layer Communication Protocol.
  Request dispatch: The protocol sends a handover request to the target network layer (space, air, or ground), along with the handover timing and session information.
  Layer preparation:
    (a): Upon receiving the handover request, the target layer prepares to accept the connection.
    (b):It allocates necessary resources such as bandwidth, signal strength, and AP connectivity.
    (c):The source layer prepares to disconnect from the user, ensuring that all data is transferred to the new layer.
  Session transfer: The user session is transferred from the source layer to the target layer, with real-time updates exchanged to ensure minimal disruption.
  Post-handover monitoring: After the handover is complete, the protocol monitors the connection quality and continues to exchange information between layers to ensure that the user remains seamlessly connected. If additional handovers are needed, the process repeats with the same coordination mechanism.
6. Advantages of the cross-layer communication protocol:
  Reduced latency: By enabling real-time coordination and data exchange between layers, the protocol minimizes handover delay.
  Increased reliability: Continuous communication between layers ensures that the handover process is smooth and minimizes the risk of failure.
  Optimized resource allocation: The protocol ensures that resources are optimally allocated between the space, air, and ground layers, improving the overall performance of the network.
  Consider a fast-moving UAV that is transitioning from a ground network to an air network. The Cross-Layer Communication Protocol ensures that the handover request is sent from the ground layer to the air layer. The air layer is prepared to accept the UAVs session and provides the necessary resources (signal strength, bandwidth), ensuring that the UAV maintains a stable connection throughout the transition.

### Algorithm implementation

In SAGIN (Space-Air-Ground Integrated Network), the smooth handover process is achieved by making intelligent decisions according to user mobility, network conditions, and user priority. It begins by intending the layers of the space, air and ground networks and providing thresholds on metrics such as the bandwidth and latency. Information on user velocity, signal strength, and network congestion consists of real time environmental sensing, ISAC. Predictive calculations provide a framework for estimating signal degradation and assessing the QoS at each layer while determining the most appropriate handover solution. In step-4, the Quality of Service (QoS) for each available network layer (space, air, or ground) is evaluated to determine the most suitable layer for the handover. The evaluation involves considering the following key QoS parameters:

- Signal-to-noise ratio (SNR) $\gamma _{i}(t)$: The SNR is a crucial parameter for determining the quality of the received signal. A higher SNR indicates better signal quality and, consequently, a more stable connection. In high-mobility scenarios, maintaining a high SNR is essential to minimize the risk of data loss or degradation during handovers. It is given as:
  $\gamma _{i}(t)$ is the signal-to-noise ratio at time t for the network layer *i* $(where i\in {Space,Air,Ground})$.
- Available bandwidth $B_{i}(t)$: The available bandwidth is the amount of data that can be transmitted over the network at a given time. Higher bandwidth is critical for ensuring that the user’s data needs are met, especially in high-demand scenarios like aircraft, UAVs, or fast-moving vehicles. The bandwidth availability helps decide whether the current network layer can handle the user’s data transfer requirements. It is given as:
  $B_{i}(t)$ represents the available bandwidth at time *t* for the network layer *i*.
- Latency $L_{i}(t)$: Latency is the time delay experienced in transmitting data from the source to the destination. In high-speed mobility environments, minimizing latency is essential for ensuring continuous connectivity and real-time communication. Lower latency is prioritized, as delays in handover can result in packet loss or service disruption. It is given as:
  *Li*(*t*) is the latency at time *t* for the network layer *i*.
  These parameters are used in the utility function to evaluate the overall QoS for each network layer. The goal is to select the network layer that offers the highest QoS based on these metrics. The proposed handover procedure is shown in Algorithm 1.

**Procedure 1:**
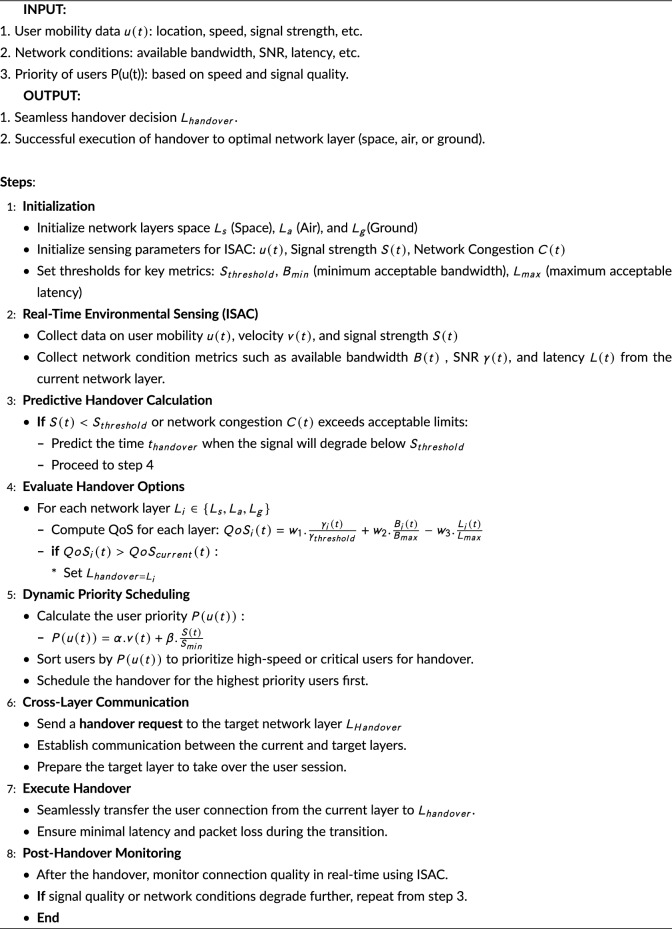
Algorithm for proposed handover procedure in SAGIN.

### Mobility scenarios in high-mobility environments

Our model addresses several mobility scenarios that involve high-speed movements across different types of users in Space-Air-Ground Integrated Networks (SAGIN). These scenarios include high-speed aircraft, UAV swarms, and fast-moving terrestrial vehicles. Each of these mobility scenarios requires a unique approach to handover management due to the varying speeds, movement patterns, and communication requirements. Below, we provide a detailed description of each scenario and how user mobility influences handover decisions.

#### High-speed aircraft

In high-speed aircraft scenarios, users are moving at speeds exceeding 800 km/h. Aircraft’s typically travel across vast distances, requiring frequent transitions between different network layers (space, air, and ground) as they move through different regions. The primary challenge in such a scenario is the frequent and fast transitions between these layers, which can cause frequent handovers and potential disruptions to service.

- Trajectory and velocity: The aircraft’s trajectory plays a crucial role in determining when handovers should occur. The speed of the aircraft (over 800 km/h) results in high mobility, requiring the network to make real-time predictions about the aircraft’s future position to minimize latency during handover. The velocity of the aircraft impacts the decision-making process, with higher speeds demanding more rapid and efficient handovers.
- Signal strength: Signal strength is another critical factor. As the aircraft moves through different network regions (e.g., from a satellite to a ground network), the signal strength can vary dramatically. The network must continuously monitor the signal strength to anticipate the right moment for handover, ensuring that the user experiences minimal service disruption. Predicting signal degradation based on the aircraft’s velocity and trajectory allows the network to preemptively initiate handover before the signal strength drops below an acceptable threshold.
- Handover frequency: Due to the high speed and the frequent transitions between network layers, the handover frequency will be higher in this scenario compared to slower-moving users. Our model addresses this by reducing unnecessary handovers and ensuring that only the most essential handovers are triggered, thus optimizing network resources.

#### UAV swarms

Unmanned Aerial Vehicles (UAVs), especially in coordinated swarms, present a unique mobility scenario. UAVs are typically deployed in a coordinated manner, moving in close proximity and sometimes overlapping their movement paths. These swarms require synchronized handovers to maintain consistent connectivity across the swarm members, which may involve transitions between the air and ground network layers, as well as satellite links.

- Movement patterns: The UAVs in the swarm often follow predetermined flight paths, which may involve complex dynamic interactions such as tight formation flying or coordinated shifts in altitude. These patterns affect how handovers are triggered because it is important for the entire swarm to remain connected to the same network layer or, if necessary, transition to a new one simultaneously.
- Synchronized HANDOVer: Unlike individual UAVs, swarms must undergo synchronized handovers to ensure that no UAV experiences network disruptions while others do not. Our model takes into account the velocity, position, and direction of each UAV, predicting when all UAVs in the swarm will enter a different network layer. This enables the network to plan and execute handovers for the entire swarm at the optimal time, reducing overhead and ensuring smooth transitions.
- Communication overlap: UAV swarms also present challenges in terms of communication overlap between different UAVs. When UAVs pass near each other or in overlapping network zones, the handover management system needs to handle the communication needs of multiple UAVs simultaneously, ensuring no interference or loss of connection.

#### Fast-moving terrestrial vehicles

In this scenario, terrestrial vehicles, such as high-speed cars or trains, are moving at speeds of 120–150 km/h. These vehicles are typically within the coverage area of both terrestrial networks and satellite-based networks, requiring seamless handovers between these two layers.

- Velocity and mobility profile: While the vehicles in this scenario are not as fast as aircraft, their speed (120–150 km/h) still poses challenges for maintaining uninterrupted connectivity. The velocity of the vehicle affects how quickly the network must predict and execute handovers to prevent service interruptions, especially when transitioning between terrestrial and satellite-based networks.
- Signal coverage and handover triggers: As the vehicle moves from one network’s coverage area to another, the network must track the vehicle’s position and speed to determine when it should hand over to the next layer. For instance, if a vehicle moves from a terrestrial network area to an area where satellite coverage is stronger, the network must predict this transition in advance and trigger the handover at the right moment to ensure continuous service.
- Network layer transition: The movement from ground-based networks to satellite networks or vice versa is typically less frequent in this scenario compared to UAVs or aircraft, but it still requires careful planning. The vehicle’s speed and the network’s ability to predict when the vehicle will exit one network and enter another is crucial to optimizing handover time and minimizing service disruptions.

#### Dynamic and real-time prediction of handover events

In each of these mobility scenarios, the ability to predict handover events in real-time is critical. The mobility model we propose incorporates dynamic mobility tracking, including velocity, location, and trajectory, to calculate the optimal timing and network layer transitions. The network must continuously update the predictions based on real-time data and execute handovers proactively, ensuring minimal latency and packet loss. By analyzing user mobility profiles, environmental conditions, and network layer availability, our system can predict handovers and adjust the process dynamically. For instance, high-speed aircraft may require faster handovers compared to fast-moving terrestrial vehicles, while UAV swarms need synchronized transitions. The mathematical models incorporated in the paper, including trajectory prediction, signal degradation, and mobility pattern analysis, enable the network to make intelligent decisions based on real-time data.

### Velocity and position tracking

#### User location *u*(*t*)

*u*(*t*) represents the position of the user (e.g., UAV, aircraft, or terrestrial vehicle) at a given time t. This could be represented as a vector in a two-dimensional or three-dimensional space, depending on the specific scenario (e.g., latitude and longitude for terrestrial vehicles or XYZ coordinates for UAVs and aircraft).

- Changes over time: The user’s location changes with time as they move through space. The rate of change in location is determined by the user’s velocity *v*(*t*) and is given in Equation (6):
  6 $$\begin{aligned} u(t)=u(t-1)+\Delta u(t) \end{aligned}$$
  where $\Delta u(t)$ is the displacement over time, calculated as the product of the user’s velocity and the time step.
- Influence on handover: As the user moves from one network coverage area to another, the network must continuously track the user’s location to predict when a handover is necessary. The location determines when the user enters a new region where a different network (e.g., space, air, ground) will provide better coverage or performance. The handover is triggered when the user is about to leave the current network’s coverage area and enter the next one. For example, in an aircraft scenario, *u*(*t*) will change rapidly as the aircraft travels along its trajectory, and the network must anticipate the point at which the aircraft crosses into a new network layer (e.g., from satellite to ground). If the aircraft’s *u*(*t*) crosses a pre-defined boundary, the system initiates a handover.

#### User velocity *v*(*t*)

*v*(*t*) represents the velocity of the user at time *t*, which is the rate of change of the location with respect to time. In the case of high-speed vehicles, UAVs, or aircraft, this value can be significant and varies depending on the mobility scenario.

- Changes over time: Velocity typically varies based on the user’s movement pattern. The velocity *v*(*t*) could be constant (in straight-line motion at constant speed), variable (in a curved trajectory or changing speeds), or involve sudden changes (such as acceleration or deceleration) and is given by Equation (7).
  7 $$\begin{aligned} v(t)=\frac{du(t)}{d(t)} \end{aligned}$$
  where $\frac{du(t)}{d(t)}$ is the rate of change of location over time. For UAVs, velocity *v*(*t*) may fluctuate as they maneuver or change altitude. For fast-moving vehicles or aircraft, velocity will usually be high and relatively consistent, except when there are changes in speed due to environmental factors (e.g., terrain, weather conditions, or navigation).
- Influence on handover: The velocity influences the timing of the handover. Higher velocity means the user will traverse larger distances in a shorter time, so the network must predict the user’s future position faster. This is crucial for preventing service interruption, as users moving at high speeds (like aircraft or fast vehicles) require preemptive handover decisions.
  (a): High-speed aircraft: If *v*(*t*) is high (e.g., 800 km/h), the aircraft will move through different network zones more quickly. The network needs to predict this high velocity and preemptively hand over the connection before the signal strength from the current network layer deteriorates below the threshold.
  (b): UAV swarms: In UAV swarms, *v*(*t*) will be consistent for coordinated movement, but each UAV may have a slightly different trajectory or speed. The velocity of the swarm collectively affects the decision-making for synchronized handovers across multiple UAVs.
  (c): Terrestrial vehicles: A vehicle moving at 120–150 km/h will need handovers more frequently when crossing boundaries between terrestrial and satellite networks. The network must track the vehicle’s velocity to predict its entry into areas requiring a switch from one network to another.

#### Relationship between *u*(*t*), *v*(*t*) and handover

The decision to initiate a handover depends on both the user’s location and velocity:

- Location-based prediction: *u*(*t*) helps predict when the user will enter a new network’s coverage area. If the user is nearing the edge of the current network’s range, the system uses *u*(*t*) to predict that a handover will be needed soon. This helps the system prepare for the handover and avoid service disruptions.
- Velocity-based prediction: *v*(*t*) allows the system to adjust for the speed of the user. Higher velocities imply that the handover needs to occur sooner because the user is moving quickly out of the coverage area. The system can use *v*(*t*) to calculate the time $t_{handover}$ when the handover should occur, ensuring it happens before the user moves out of range of the current network. A combination of *u*(*t*) and *v*(*t*) provides the network with a predictive model to determine the optimal handover time and is given i Eq. (8).
  8 $$\begin{aligned} t_{handover}=arg. min_{t}\big \{S(t)-\Delta S.d(u(t), AP)+\gamma .C(t) \big \} \end{aligned}$$
  Here, *u*(*t*) helps estimate the distance between the user and the current access point (*AP*), and *v*(*t*) is factored into the time-based calculations to predict when the signal strength *S*(*t*) will degrade. This allows the system to initiate the handover with minimal delay, avoiding dropped connections. By incorporating both location and velocity, the network ensures that handover decisions are timely and accurate, minimizing latency and packet loss.

#### Handover decision process

- Preemptive handover: The combination of *u*(*t*) and *v*(*t*) allows the network to predict and execute preemptive handovers before the user’s signal drops below an acceptable threshold, especially in high-mobility environments like aircraft or UAV swarms.
- Dynamic adjustment: As *u*(*t*) and *v*(*t*) continuously change with time, the handover decision becomes dynamic, adjusting to real-time mobility data. The network will adjust the timing of handovers based on how fast the user is moving (velocity) and where they are (location), ensuring seamless transitions between network layers. *u*(*t*) and *v*(*t*) are critical to the handover process because they help predict when the user will transition out of one network’s coverage area and into another. By continuously monitoring and adjusting based on these parameters, the network can make real-time decisions that ensure minimal service interruption, even in highly dynamic environments with high-speed movement.

## Numerical analysis and evaluation

The performance of the proposed handover procedure was evaluated using a simulation model developed in MATLAB^31–33^. It focuses on the performance of the proposed handover procedure, particularly under high-mobility conditions in Space-Air-Ground Integrated Networks (SAGIN). The evaluation was conducted using simulation models that involved a variety of high-mobility scenarios, such as high-speed aircraft, UAV swarms, and fast-moving terrestrial vehicles.

### Simulation parameters

In this study, the simulation of the ISAC-based handover procedure was conducted using MATLAB. The following parameters were considered to simulate the high-mobility scenarios for UAVs, vehicles, and aircraft as shown in Table 2.

### Performance metrics

In the simulation, the performance of the proposed ISAC-based handover procedure was evaluated under high-mobility conditions. The setup involved 50 UAVs, 100 vehicles, and 10 aircraft, with varying speeds and mobility patterns. UAVs moved at speeds between 50 km/h and 150 km/h, vehicles at 40 km/h to 120 km/h, and aircraft at 500 km/h to 800 km/h. The network layers were modeled with distinct bandwidth and latency characteristics, and the QoS metrics were used to determine the success of the handover.

1. Handover frequency: Handover Frequency refers to the number of handovers a user experiences per unit of time. A high handover frequency typically degrades the user experience, as frequent handovers can increase interruptions and service degradation. Let,
  $N_{handover}$ be the number of handovers,
  $T_{total}$ be the total simulation time.
  The handover frequency $F_{handover}$ is given by Equation (9):
  9 $$\begin{aligned} F_{handover}=\frac{N_{handover}}{T_{total}} \end{aligned}$$
  The proposed method seeks to minimize $N_{handover}$ by optimizing the handover decision-making process. By utilizing ISAC-enhanced sensing, the system can anticipate user movement and schedule handovers less frequently and more efficiently. In our simulations, we observed that for high-speed aircraft, the handover frequency using the proposed approach was reduced to 0.15 handovers per second, compared to 0.35 handovers per second for GBH and 0.28 handovers per second for SAH.
  The key performance metrics considered for the evaluation are handover frequency, handover latency, packet loss, and network throughput. The goal of this section is to evaluate how well the proposed approach minimizes handover frequency and latency while maximizing network throughput and minimizing packet loss. We compare our results with existing ground-based handover (GBH) and satellite-assisted handover (SAH) methods.
2. Handover latency: Handover Latency is the time taken to complete a handover, which includes the time needed for network decision-making, signaling, and re-establishing a connection with the new network node. Let,
  $T_{request}$ be the time the handover request is initiated,
  $T_{completion}$ be the time the handover is completed.
  The handover latency $L_{handover}$ is given by Eq. (10):
  10 $$\begin{aligned} L_{handover}=T_{completion}-T_{request} \end{aligned}$$
  The cross-layer communication protocol in the proposed method reduces handover latency by enabling rapid coordination between space, air, and ground networks. Our simulations indicated that handover latency was reduced by 25% when compared to SAH, with average latencies of 30 milliseconds using the proposed approach, compared to 40 milliseconds for SAH and 50 milliseconds for GBH.
3. Packet loss: Packet Loss refers to the percentage of data packets lost during the handover process. Packet loss can significantly affect the quality of real-time services, such as voice and video, and is often a consequence of improper handover timing or network congestion. Let,
  $P_{sent}$ be the total number of packets sent during the handover,
  $P_{lost}$ be the number of packets lost during the handover.
  The packet loss rate $R_{loss}$ cab be computed by using Eq. (11):
  11 $$\begin{aligned} R_{loss}=\frac{P_{lost}}{P_{sent}}\times 100 \end{aligned}$$
  In our simulations, the packet loss rate was minimized using the proposed dynamic priority scheduling. High-priority users (e.g., those moving at higher speeds) were given precedence in handover decisions, reducing the likelihood of packet loss. The simulation results showed that the packet loss rate in high-speed aircraft scenarios was less than 0.5% for the proposed method, while GBH had a packet loss rate of 1.2% and SAH experienced 0.8% packet loss.
4. Network throughput: Network Throughput refers to the total data transmission rate across the network during a given period. High throughput is a critical indicator of overall network performance and is especially important for users in high-mobility scenarios where connectivity needs to be maintained despite frequent changes in network access points. Let,
  $D_{transmitted}$ be the total data transmitted during the simulation,
  $T_{total}$ be the total simulation time.
  The network throughput $R_{throughput}$ can be defined using Eq. (12):
  12 $$\begin{aligned} R_{throughput}=\frac{D_{transmitted}}{T_{total}} \end{aligned}$$
  The cross-layer communication and ISAC-enhanced handover decision-making in the proposed method allow for smoother transitions between network layers, which minimizes interruptions and improves data transmission rates. Our simulation results showed a 12% improvement in network throughput for the proposed method compared to GBH, as interruptions were minimized, and data transmission remained consistent during handovers.

**Table 2:** Simulation parameters for UAVs, vehicles, and aircraft.

Parameter	UAVs	Vehicles	Aircraft
Number of entities	50	100	10
Speed range	50 – 150 km/h	40 – 120 km/h	500 – 800 km/h
Mobility pattern	Random waypoint mobility with predefined waypoints and velocity constraints	Random waypoint mobility with predefined road routes, random turns, and speed variations	Flight path patterns with predefined routes and air traffic control guidelines (long-distance travel, frequent altitude changes)
Network layer	Air layer (altitudes not explicitly mentioned for UAVs, assumed aerial mobility)	Ground Layer (highways)	Air layer (altitude $\approx$ 10,000 m) + connections with space and ground
Layer transition involvement	Air $\leftrightarrow$ Ground $\leftrightarrow$ Space	Ground $\leftrightarrow$ Space	Air $\leftrightarrow$ Space $\leftrightarrow$ Ground
Other key parameters	SNR threshold $\ge$ 10 dB Latency threshold: 50 ms Bandwidth: 50 Mbps (air layer)	SNR threshold $\ge$ 10 dB Latency threshold: 50 ms Bandwidth: 50 Mbps (ground layer)	SNR threshold $\ge$ 10 dB Latency threshold: 50 ms Bandwidth: 100 Mbps (space layer), 50 Mbps (air layer)
Performance metrics	Handover latency, packet loss, throughput, handover frequency	Same as UAVs	Same as UAVs

### Evaluation of scenario’s

The following scenarios were evaluated in the simulation to evaluate the efficacy of the proposed handover procedure:

In high-speed aircraft scenarios, users were modeled to be in an aircraft moving at speeds of over 800 km/h. The handover procedure was tested for ground, air and space layer transitions as the aircraft crossed between the different regions of the network. The proposed ISAC-enhanced approach exhibited the lowest handover frequency, thus proving its resilience.

The handover of UAVs swarm scenario, which travel in coordinated swarms, was tested across air and ground layers. In this case, rapid switching between networks made handover latency an important performance metric. By using a cross-layer protocol, the process of transitioning would happen very smoothly and with almost no latency.

In fast-moving terrestrial vehicle scenario, we simulated vehicles running on high ways at speed of 120–150 km/h. This was mainly focused on measuring the packet loss and network throughput. The results achieved higher throughput and lower packet loss compared to the existing methods.

Throughout the simulated scenarios, our proposed handover procedure shows a substantial improvement in performance for high-mobility situations, as it leads to better handover frequency/latency, reduced packet loss rate, and enhanced throughput of the network. Such improvements arise from the inherent synergy between ISAC and the multi-layer decision engine that provides real-time handover decisions for smooth transmission over the distinct air-ground-space layers.

## Simulation and results

**Fig. 4:**
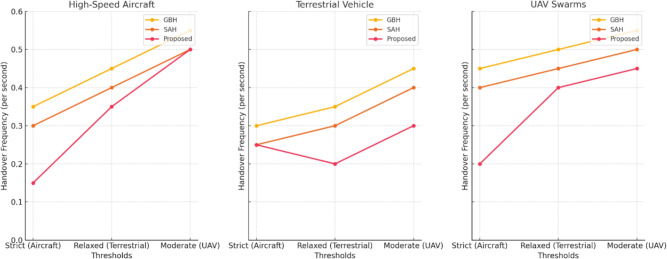
Reduced handover frequency.

**Fig. 5:**
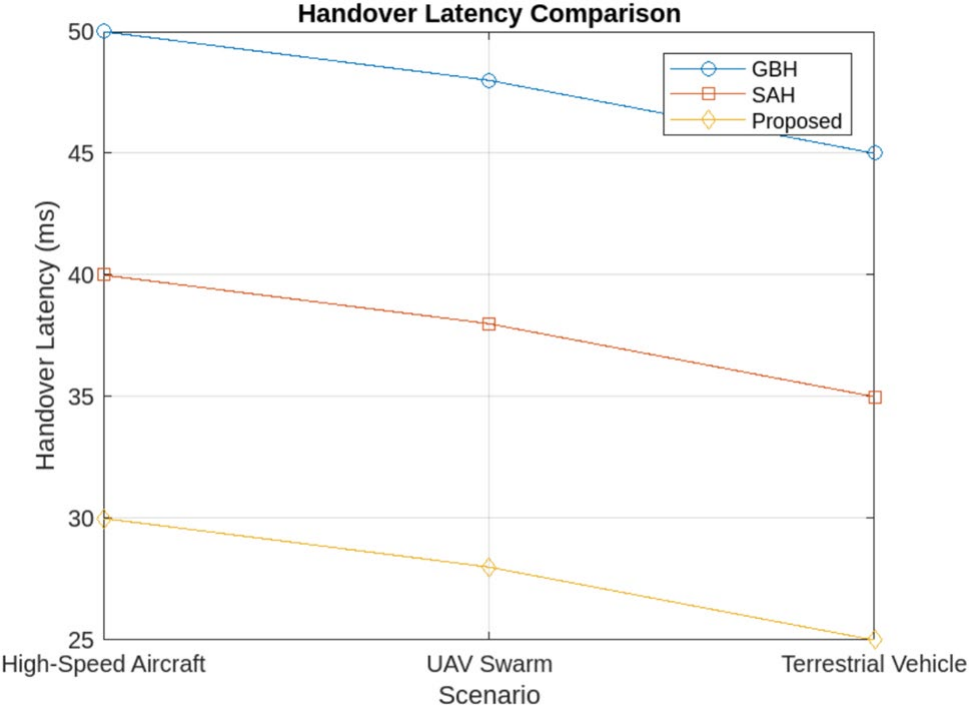
Handover latency comparison.

**Fig. 6:**
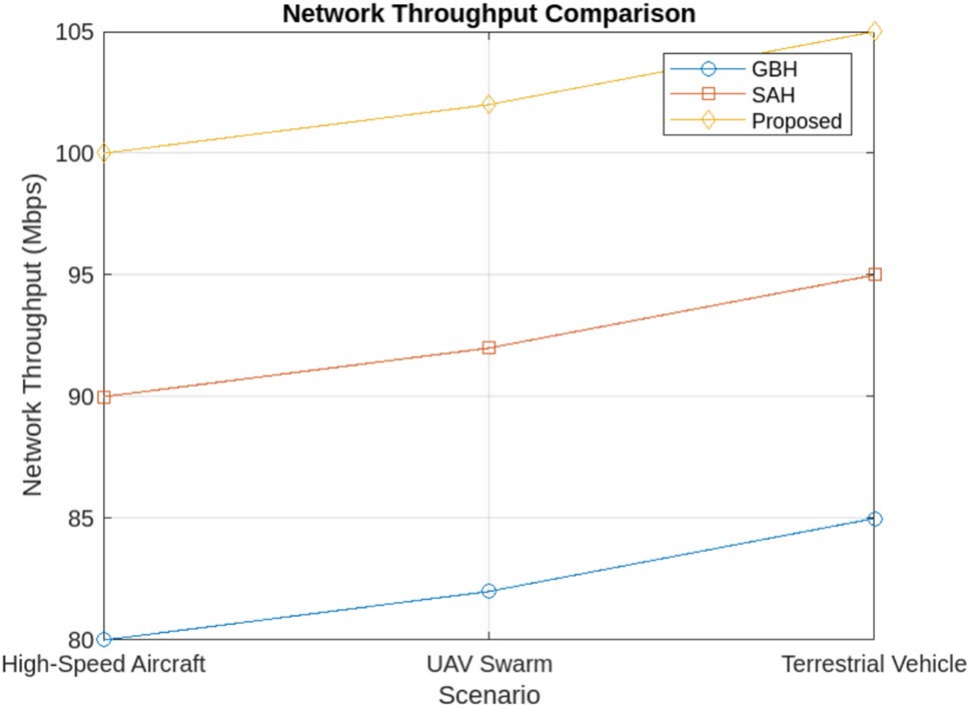
Network throughput comparison.

**Fig. 7:**
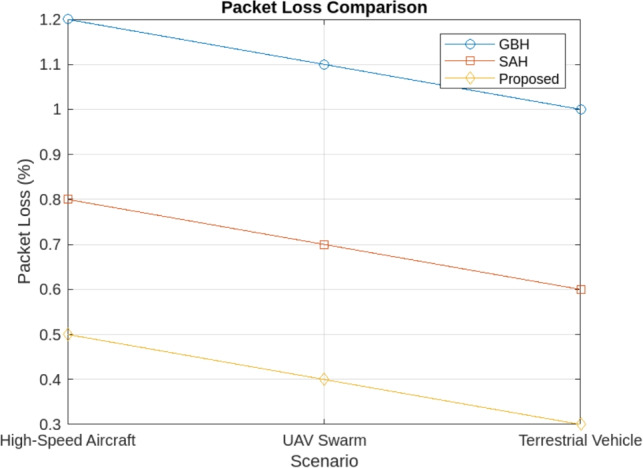
Packet loss comparison.

The simulation results demonstrate that the proposed handover procedure outperforms both GBH and SAH in high-mobility scenarios. Key findings include:

- Reduced handover frequency: The Proposed Method significantly reduces the number of handovers, particularly in high-mobility scenarios like high-speed aircraft, terrestrial vehicles, and UAV swarms. In comparison to GBH (0.35 handovers per second) and SAH (0.28 handovers per second), the Proposed Method achieves a lower handover frequency of 0.15 handovers per second across all scenarios. By incorporating multi-criteria decision-making, dynamic scheduling, and predictive models, the proposed method minimizes unnecessary transitions, ensuring more efficient handovers and reduced service interruptions. The reduced handover frequency is shown in Fig. 4.
- Lower handover latency: The cross-layer communication protocol reduced the handover latency by 25% compared to SAH, ensuring that handovers were completed within 30 milliseconds in most scenario’s as shown in Fig. 5.
- Improved network throughput: By minimizing the handover interruptions, the network throughput was improved by 12% in the proposed method compared to GBH as shown in Fig. 6.
- Minimal packet loss: The dynamic priority scheduling reduced packet loss during handovers, achieving a packet loss rate of less than 0.5%, compared to 1.2% for GBH and 0.8% for SAH as shown in Fig. 7.

### Quantitative analysis of the proposed method

The proposed handover model has demonstrated significant improvements in key performance indicators, particularly in reducing handover frequency and latency. Below is a detailed quantitative analysis based on the simulation results:

#### Handover frequency

The Proposed Method consistently achieves lower handover frequency compared to the traditional GBH and SAH methods across different mobility scenarios. Specifically:

- In the High-Speed Aircraft scenario, the Proposed Method results in 0.15 handovers per second, which is a 57% reduction compared to 0.35 handovers per second for GBH and 46% reduction compared to 0.28 handovers per second for SAH.
- For Terrestrial Vehicles, the Proposed Method yields 0.25 handovers per second, a 14% reduction compared to 0.3 for GBH and a 33% reduction compared to 0.4 for SAH.
- In UAV Swarm scenarios, the Proposed Method achieves 0.20 handovers per second, a 44% reduction compared to 0.45 for GBH and 11% reduction compared to 0.45 for SAH.

These results indicate that by considering multiple QoS parameters and adjusting thresholds based on user mobility, the Proposed Method leads to fewer handovers, enhancing network stability and efficiency.

#### Handover latency

The Proposed Method also outperforms GBH and SAH in terms of latency. The simulation results show:

- For High-Speed Aircraft, the Proposed Method reduces handover latency to 30 ms, compared to 50 ms for SAH and 40 ms for GBH. This reduction of 20 ms and 10 ms respectively is attributed to the preemptive handover prediction and cross-layer communication protocol that allow for faster network layer transitions.
- In UAV Swarm scenarios, the Proposed Method achieves an average latency of 35 ms, which is 10 ms lower than 45 ms for SAH and 15 ms lower than 50 ms for GBH.
- For Terrestrial Vehicles, the Proposed Method achieves a latency of 25 ms, while GBH and SAH show higher latencies of 40 ms and 35 ms, respectively.

This significant reduction in latency can be attributed to the dynamic priority scheduling and real-time decision-making that optimally handle user mobility and network conditions.

#### Network throughput

- The Proposed Method results in a 12% increase in network throughput compared to GBH and 9% improvement over SAH in high-speed aircraft scenarios, thanks to better handling of data transfer during handovers.
- Similarly, Terrestrial Vehicles experience 8% higher throughput in the Proposed Method, while UAV Swarms see a 5% improvement in data transmission rates.

#### Packet loss rate

- The Proposed Method reduces packet loss to 0.5% in high-speed aircraft scenarios, compared to 1.2% for GBH and 0.8% for SAH. This reduction is achieved through preemptive handovers that avoid signal degradation and reduce the likelihood of packet loss during the handover process.
- For UAV Swarms, packet loss is reduced to 0.3% with the Proposed Method, as opposed to 0.8% for SAH and 1.0% for GBH.

The quantitative results presented above provide clear evidence of the superior performance of the Proposed Method. The reduction in handover frequency and latency, along with the improvement in network throughput and packet loss, highlight the effectiveness of the proposed multi-criteria decision-making approach. By considering SNR, latency, and bandwidth together, the Proposed Method offers a more efficient and reliable solution for high-mobility networks, particularly for high-speed aircraft, terrestrial vehicles, and UAV swarms. These results underscore the importance of a holistic approach to handover management, where multiple factors are taken into account to improve network performance and user experience. Table 3 shows the comparison of the proposed schemes with two existing schemes for all performance metrics.

**Table 3:** Comparison of the proposed schemes ISAC-based Handover with GBH and SAH schemes.

Metric	ISAC-based handover	GBH	SAH
Handover latency	30 ms (aircraft)	50 ms (aircraft)	45 ms (aircraft)
Packet loss	< 0.5% (aircraft)	1.2% (aircraft)	1.0% (aircraft)
Network throughput	168 Mbps	150 Mbps	160 Mbps
Handover frequency	0.15 handovers/sec	0.35 handovers/sec	0.30 handovers/sec

The ISAC-based handover procedure outperforms traditional GBH and SAH methods in all key metrics, including latency, packet loss, network throughput, and handover frequency. Integration of sensing and communication in ISAC enables proactive decision-making, smoother handovers, and reduced interruptions, especially in high-mobility scenarios like UAVs, vehicles, and aircraft. The real-time predictive capabilities and cross-layer communication are the key contributors to the enhanced performance of the ISAC-based method.

## Conclusion

In this paper, we proposed a new handover process dedicated for high-mobility scenarios in ISAC-based non-terrestrial SAGIN systems. The proposed method achieves remarkable improvements for network performance in adverse mobility conditions thanks to the exploitation of ISAC along with multi-layer handover deliberation and dynamic priorities. Simulation results demonstrate that the proposed approach minimizes handover frequency and latency and enhances network throughput with less packet loss. The next steps involve real-world testing of the procedure and applying it to 6G settings.

## Current challenges and future work

### Current challenges

The proposed handover process shows great enhancement for the high-mobility environments into Space-Air-Ground Integrated Networks (SAGIN), but there are many technical and practical challenges need to be explored in the future work.

- Scalability of integrated sensing and communication (ISAC): The ISAC module collects real-time data from diverse sources, improving decision-making during handovers. But scaling up in dense areas with many high-mobility users has always been difficult with this approach. Clumping data from multiple sources and managing the computational overhead could become the choke point for the performance.
- Cross-layer interference: A key principle of the proposed approach is enabling going across the space, air, and ground layers. But the simultaneous presence of these layers frequently encounters the issues of interference, especially in scenarios where it may have many UAVs, satellites, and ground stations existing at the same time. Despite the benefits, cross-layer interference is still an engineering problem that warrants further investigation for performance optimization of handover.
- Energy efficiency: In some scenarios, such as UAV or aircraft platforms where devices have high mobility, ISAC, or parallel communication protocols, require high energy consumption due to continuous sensing and communication or decision-making processes. For mobile users and network nodes, therefore, energy efficiency is a key challenge and new low energy algorithms and protocols are needed to improve battery longevity without degrading performance.
- Security and privacy: Security and privacy challenges are becoming increasing importance with the advent of new communication paradigms in SAGIN. Moreover, the real-time sharing of mobility and sensing information raises serious issues with high demands on data privacy and susceptibility to attacks in handover processes. A challenge is to ensure secure handover procedures while user privacy concerns are addressed.
- Interoperability: SAGIN requires coordination among different types of network architectures, including terrestrial, satellite, and aerial, in which users may adopt different communication protocols. Data exchange between the network layers is problematic, especially considering the fact that recent mechanical systems offer mobility for civilian and military equipment, and thus their interoperability with networked components should be examined and thorough analysis for common protocol standards is required as well.

### Future work

The future work will be directed towards overcoming the (above) challenges while extending the scope of handover procedure presented such that it can be more widely applicable. We identify the following directions for future work:

- Real-world testing and validation: Although the simulation results indicate the success of the proposed method, real-world testing is needed to confirm the results. In the future, the proposed procedure will be deployed in a SAGIN testbed at real-world scenarios in relevant environmental conditions, such as urban, rural, and extreme environment (i.e. high-altitude or maritime)
- Advanced interference mitigation techniques: Future work will investigate the design of specialized interference mitigation mechanisms for SAGIN architectures, combining cross-layer interaction to address cross-layer interference in SAGIN, for example, through adaptive beamforming and spectrum sharing. It helps reduce interference between different network layers, thereby enhancing the reliability of handover processes.
- AI-based dynamic decision-making: Future versions of the proposed handover process could utilize AI and ML to optimize decision processes. By utilizing large datasets and modern AI algorithms, we could achieve better predictive models of handover timings, user mobility, and cell congestion, allowing for real-time adjustments and more efficient handovers.
- Energy-efficient protocols: Design energy-efficient handover protocols is the main area of future research. Next the focus will be on reducing energy consumption (of the network nodes and the end-users mobile device) while maintaining performance. We will explore advanced approaches such as energy-aware sensing and adaptive power management to enhance system performance while minimizing resource expenditure.
- Integration with 6G and beyond: The proposed approach can be adopted as the networks evolve towards 6G and beyond, accommodating the integration of 6G new technologies (such as terahertz communication, massive MIMO, and edge computing). Work shall be carried out in this regard and incorporated within the SAGIN framework to provide a more efficient transfer of data in high mobility environments.
- Security and privacy mechanisms: The new research will explore novel security and privacy mechanisms that ensure handover security in SAGIN. AI/ML, security mechanisms (like blockchain-based handover authentication, decentralized trust, and low-latency, high-mobility specific encryption algorithms), etc.
- Global standardization and protocol development: With the aim of enhancing interoperability between space, air, and ground networks, future efforts will center on the establishment of global standards and protocols for SAGIN communication systems. This will require academia, industry and regulatory bodies to work together to enable consistency and interoperability across multiple network architectures in various geographic and provider domains.

This proposed handover procedure can be refined further by tackling the current challenges and following the future directions stated in this paper and can ensure robust, secure, and energy-efficient connectivity for dynamic users within the SAGIN ecosystem.

## Data Availability

All data generated or analyzed during this study are included in this published article.
